# Therapeutic Approaches Targeting Protein Aggregation in Amyotrophic Lateral Sclerosis

**DOI:** 10.3389/fnmol.2020.00098

**Published:** 2020-06-09

**Authors:** Ravinder Malik, Martina Wiedau

**Affiliations:** Department of Neurology, David Geffen School of Medicine, University of California, Los Angeles, Los Angeles, CA, United States

**Keywords:** Lou Gehrig’s disease, protein misfolding, superoxide dismutase, C9ORF72 DPRs, motor neuron disease, proteinopathies, proteostasis

## Abstract

Amyotrophic lateral sclerosis (ALS) is a debilitating neurodegenerative disease that targets motor neurons (MNs) in the brain and spinal cord. It leads to gradual loss of motor signals to muscles leading to atrophy and weakness. Most patients do not survive for more than 3–5 years after disease onset. Current ALS treatments provide only a small delay of disease progression. Therefore, it is of utmost importance to explore new therapeutic approaches. One of the major hindrances in achieving this goal is poor understanding of causes of the disease. ALS has complex pathophysiological mechanisms in its genetic and sporadic forms. Protein aggregates are a common hallmark of ALS regardless of cause making protein pathways attractive therapeutic targets in ALS. Here, we provide an overview of compounds in different stages of pharmacological development and their protein pathway targets.

## Introduction

Amyotrophic lateral sclerosis (ALS) or Lou Gehrig’s disease is the most common motor neuron (MN) disease. It is a progressive and fatal neurodegenerative disease, which affects both upper MNs in the motor cortex and lower MNs in brain stem and the anterior spinal cord (Ravits and La Spada, 2009). ALS patients experience muscle cramps, fasciculations, progressive muscle atrophy and weakness, hyperactive reflexes, difficulty with speech, chewing, and swallowing. Ultimately, the breathing muscles are affected leading to respiratory failure (Borasio and Miller, 2001). The average age of disease onset is 55 years (Naruse et al., 2019). More than half of all patients do not live more than 3–4 years after diagnosis, 20% live 5 years or more, and only 10% live more than 10 years. Men are at a 1.2 times higher risk to get the disease as compared to women. Other possible risk factors include genetics, aging, and environmental factors such as toxins, metals, smoking, traumatic head injury, and infections (Seals et al., 2016a; Spencer et al., 2019). Military veterans are approximately twice as likely to develop ALS compared to the average prevalence (Seals et al., 2016b). So far only two drugs are approved by the Federal Drug Administration (FDA) to treat ALS: riluzole and edaravone. These drugs provide limited relief and slow disease progression by a few months. There is no known therapy that can halt the disease. The exact mechanism of the disease is still unknown. Research studies suggest that multiple phenomena can be involved such as protein misfolding and aggregation, impairment of protein trafficking, oxidative stress, RNA dysmetabolism, failure of protein clearance machinery, and imbalance in protein homeostasis (Blokhuis et al., 2013; Parakh and Atkin, 2016; Ramesh and Pandey, 2017; McAlary et al., 2019). Sporadic forms comprise about 90–95% of all cases. Familial ALS (fALS) accounts for 5–10% of all cases in the United States (Mehta et al., 2018) involving genes such as superoxide dismutase 1 (SOD1), Chromosome 9 open reading frame 72 (C9ORF72), tar-DNA binding protein 43 (TDP-43), and fused in sarcoma (FUS) (Chen et al., 2013; Brenner and Weishaupt, 2019).

Protein aggregation is an important feature of ALS pathology. Amyloid deposits from different proteins such as TDP-43, C9ORF72 dipeptide repeats (DPRs), phosphorylated high molecular weight neurofilament protein (pNFH), rho guanine nucleotide exchange factor (RGNEF), and FUS have been detected in ALS MNs (Blokhuis et al., 2013). These aberrant protein deposits may be toxic to the cells, leading to neurodegeneration and are potential targets for therapeutic interventions. In this review, we focus on proteins which form MN aggregates implicated in ALS pathology. Multiple promising efforts to therapeutically target these proteins, either by specific approaches, e.g., interaction modulators or through small molecules or via indirect and/or wide-ranging methods, e.g., by protein degradation pathway regulators or by antisense, are highlighted. We discuss examples of emerging and promising therapeutic candidates at their different stages of development (Table 1).

**TABLE 1:** Therapeutic protein aggregation targets in ALS.

Therapeutic candidate	Target	Process affected/checked
AAV9-ShRNA-SOD1	SOD1	Suppression of mutant SOD1 mRNA
Arimoclomol	Aggregated proteins	Enhance expression of heat-shock proteins, involved in clearance of aggregated protein
BIIB078	C9ORF72 gene	Antisense against C9ORF72 mRNA to blocks it translation
Colchicine	Proteasome and autophagy	Promotes expression of HSPB8 and autophagy-mediated removal of misfolded proteins
Macrophage migration inhibitory factor (MIF)	SOD1	Misfolding of SOD1
Molecular Tweezer (CLR01)	SOD1	Targets the self-assembly of SOD1
Myricetin	Aggregated proteins	Clearance of protein aggregates by upregulation proteasomal degradation mechanisms
Recombinant human monoclonal antibody (α-miSOD1)	SOD1 Aggregates	Misfolded Sod 1
Single-chain variable fragment (scFv) derived from the 3B12A monoclonal antibody (MAb)	TDP-43	TDP-43 nuclear export signal
Single-chain variable fragment (scFv) named VH7Vk9	TDP-43	Binding of RNA recognition motif 1 (RRM1) of TDP-43
tgG-DSE2lim and tgG- DSE5b	SOD1	Vaccine against early unfolded protein for rapid clearance by immune system
Tofersen (BIIB067)	SOD1	Reduction in SOD1 protein level by antisense

## Defining Protein Aggregation

**FIGURE 1:**
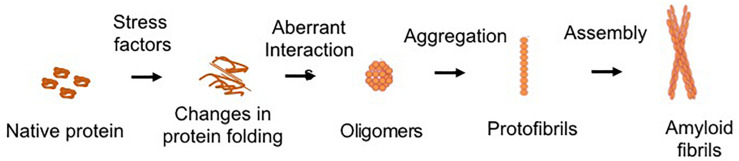
Schematic of common protein aggregation pathway. The biologically active native form of protein can undergo changes in its structure which lead to unfolding or misfolding. The hydrophobic motifs interact to form the oligomeric structures. Larger aggregates of oligomers lead to the formation of protofibrils, which assemble to form the mature fibrillar structures.

Protein aggregation is the process of aberrant folding of a protein leading to self-association that may cause the formation of oligomers and fibrils via polymerization. The resulting amyloid fibrils are rich in beta sheet structure. Physiologically, proteins undergo folding through chaperones to attain their biologically favored stable conformation. Protein structure is stabilized by covalent and non-covalent interaction between the amino acids (Dobson, 2003). In disease, structural destabilization leads to exposure of hydrophobic amino acids in the outer environment, which have an affinity to self-assemble into larger aggregates and fibrils. The basic mechanism of protein aggregation is through a misfolded or unfolded conformation of the protein monomer that leads to the exposure of hydrophobic patches into hydrophilic cellular environment (Figure 1). The adhesive nature of these patches may lead to self-association into oligomers and eventually into fibrils implicated in many neurodegenerative diseases as non-specific interactions with other cellular proteins interfere with normal cell functions. The intermediates and oligomers can interact with cellular membranes harming membrane integrity leading to the leakage of cellular material and cell death (Holmes et al., 2014).

**FIGURE 2:**
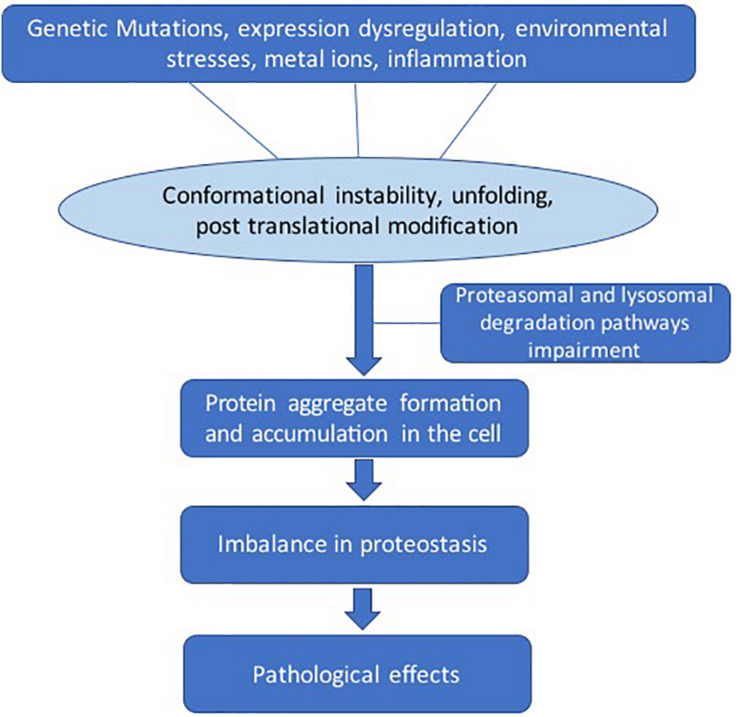
General pathways involved in pathological protein aggregation. Several factors can play a role such as genetic mutation, protein expression dysregulation, environmental effects, metal ions, and inflammation. These conditions can lead to several changes such as conformation instability, unfolding, and post-translational modifications. The clearance mechanism and quality control pathways fail to maintain proteostasis.

There are several factors that lead to destabilization of protein structure (Figure 2). Genetic mutations can change the amino acid sequence, leading to altered protein conformation. Dysregulation of molecular chaperone network functions, which govern the protein quality control processes such as protein unfolding and disaggregation and targeting terminally misfolded proteins for proteolytic degradation (Kim et al., 2013). Environmental effects such as changes in pH, temperature, infection, or chemical modification can also destabilize proteins. The larger protein aggregates accumulate in the cell or extracellularly if clearance mechanisms fail (Rubinsztein, 2006). The dysregulation in the regulatory mechanisms of the cells can lead to imbalance in the homeostasis of proteins (proteostasis) (Labbadia and Morimoto, 2015).

The neurodegenerative diseases caused by protein aggregation are termed proteinopathies. Protein aggregates are the hallmarks of several neurodegenerative disease, for example, amyloid beta and tau in Alzheimer’s disease, alpha-synuclein in Parkinson’s disease (PD), and huntingtin in Huntington’s disease (Forman et al., 2004). ALS is a complex disease as multiple aggregating proteins such as SOD1, TDP-43, FUS, pNFH, and others have been linked to the disease (Chen et al., 2013; Brenner and Weishaupt, 2019).

## Therapeutic Targets Involving Protein Aggregates in ALS

### Targeting Protein Aggregates by Antibodies or Their Derived Fragments

#### α-miSOD1

A recombinant human monoclonal antibody (MAb) (α-miSOD1) was generated by screening human memory B cells from a large cohort of healthy elderly subjects. This antibody selectively binds to misfolded SOD1, but not to physiological SOD1 dimers. On postmortem spinal cord sections from 121 patients with ALS, α-miSOD1 antibody identified misfolded SOD1 in a majority of cases, regardless of their SOD1 genotype. In transgenic mice overexpressing disease-causing human SOD1-G37R or SOD1-G93A mutations, treatment with the α-miSOD1 antibody delayed the onset of motor symptoms, extended survival by up to 2 months, and reduced aggregation of misfolded SOD1 and MN degeneration (Maier et al., 2018).

#### Single-Chain Variable Fragment of Antibodies

TDP-43 is a ubiquitous protein encoded by the tar-DNA binding protein 43 gene. It is an essential gene for the development of the CNS from the earliest stages of embryonic life to adulthood. TDP-43 is associated with multiple steps of transcriptional and post-transcriptional regulation. Under physiological conditions, the majority of TDP-43 is nuclear, while a small proportion is continuously involved in nucleocytoplasmic relocation and may form aggregates in the cytoplasm (Baloh, 2011). Aggregates of wild-type TDP-43 are present in both sporadic and familial cases of ALS. Reduction in cytoplasmic TDP-43 inclusions is a promising strategy for both types of ALS. Clearance of TDP-43 was targeted using single-chain variable fragment (scFv) derived from the 3B12A MAb which can recognize a specific region of TDP-43 nuclear export signal called D247. HEK293A cells were transfected with tagged mutant TDP-43 plasmids. Using 3B12A scFv, mislocalized TDP-43 was detected to have a defective nuclear localizing signal. 3B12A scFv also accelerated proteasome-mediated degradation of aggregated TDP-43, most probably due to an endogenous proline (P), glutamic acid (E), serine (S), and threonine (T) rich sequence (PEST). Addition of the chaperone-mediated autophagy related signal to 3B12A scFv induced HSP70 transcription, which further enhanced TDP-43 aggregate clearance and cell viability. The 3B12A scFv reduced TDP-43 aggregates in embryonic mouse brain after *in utero* electroporation without any apparent side effects in postnatal brain pathology or development (Tamaki et al., 2018). In a similar approach, scFv antibody VH7Vk9 was produced against the RNA recognition motif 1 (RRM1) of TDP-43, which is responsible for abnormal protein self-aggregation and interaction with p65 NF-κB (Buratti, 2015). Virus-mediated delivery of VH7Vk9 in HEK293 cells and TDP-43 mice resulted in reduction of the cytoplasmic/nuclear TDP-43 ratio. Colocalization of TDP-43 with ubiquitin and microtubule associated protein 1A/1B light chain (LC-3) suggested improved clearance of the protein via proteasome and autophagosomes. Contralateral and ipsilateral cortices showed reduced microglial activation in TDP-43 mice with improvements of motor functions and cognitive deficits (Pozzi et al., 2019).

### Targeting Protein Aggregates by Vaccines

The seeding hypothesis suggests that protein aggregates can spread the disease pathology to adjacent cells and brain regions. Recent reports have indicated that misfolded SOD1 can act like prions and spread the disease (Healy, 2017; Sibilla and Bertolotti, 2017). Exogenously administered non-native misfolded or aggregated protein has been found to generate an immune response (Malik and Roy, 2011). Even if the exogenous protein is human in nature, the immune system recognizes it as foreign due to its non-native protein conformation. In this approach, the host system would generate antibodies against aggregated protein and clear them through immune response. One study in hSOD1-G37R transgenic mice used an immunological therapy with misfolded protein. Two ALS vaccines against unfolded SOD1, tgG-DSE2lim and tgG-DSE5b, were investigated (Zhao et al., 2019). Both vaccines showed rapid, robust, and well-sustained epitope-specific antibody responses and increased the life span of treated animals. The question still remains how successfully this approach can be modified to address different mutants and conformations of SOD1 protein. The challenge is to generate antigens for multiple possible conformations of the aggregated protein which is difficult due to transient nature of certain conformations of protein aggregates.

### Targeting Self-Assembly Process by Small Molecules

#### Molecular Tweezers

Small horseshoe shaped molecules termed molecular tweezers (MTs) bind reversibly to specific amino acid residues of proteins which enables targeting the process of aggregation rather than targeting a specific protein or protein conformation. MTs achieve this activity by hydrophobic and electrostatic interactions involving labile binding to positively charged amino acid residues, primarily lysine and to a lower extent arginine. Hydrophobic and electrostatic interactions are important, particularly in the early stages of the aberrant self-assembly process which are effectively interrupted by MTs (Malik et al., 2019). They do not affect the protein’s bioactivity, but aberrant interactions leading to protein aggregation can be prevented. Using purified recombinant wild-type and mutant SOD1, it was found that the lead MT, CLR01, inhibited the *in vitro* aggregation of different isoforms of SOD1. In a SOD1-G93A transgenic mouse model, CLR01 treatment decreased misfolded SOD1 in the spinal cord significantly. A small, dose-dependent decrease in disease duration was found in CLR01-treated, compared to vehicle-treated animals, yet motor function did not improve in any of the treatment groups (Malik et al., 2019). The MT has been shown to be effective against multiple proteins (Malik et al., 2019) and could, thus become an ideal therapeutic candidate for ALS with its known aggregates of multiple proteins.

### Targeting Proteasome and Autophagy

#### Colchicine

Colchicine is a plant alkaloid that interrupts microtubule formation and other cellular processes. This compound enhances the expression of heat-shock protein B8 (HSPB8) and several other autophagy factors (Crippa et al., 2016). HSPB8 recognizes and promotes the autophagy-mediated removal of misfolded mutant SOD1, as well as TDP-43 fragments from MNs and aggregating species of dipeptides produced in C9ORF72-related diseases (Crippa et al., 2016). A Phase II randomized double-blind, placebo-controlled clinical trial with colchicine in ALS (Co-ALS) was recently initiated. ALS patients will be enrolled in three groups—placebo, colchicine 0.01 mg/day, and colchicine 0.005 mg/day (Mandrioli et al., 2019). The trial will assess safety, tolerability, respiratory function, and functional ratings scale in ALS patients. The investigators will also study the cellular effects of colchicine on specific processes such as autophagy, protein aggregation, stress granules, and exosome secretion. A parallel biomarker analysis of neurofilament protein expression will be performed (Mandrioli et al., 2019).

#### Arimoclomol

An investigational drug candidate, arimoclomol enhances expression of HSPs. Previous studies of transgenic SOD1 mice showed a large safety margin up to 300 mg/day. In separate studies, the effect of arimoclomol was tested in early and late stages of the disease. The compound showed promising results in both disease stages as survival was improved (Kieran et al., 2004; Kalmar et al., 2008). A double-blind, placebo-controlled trial was initiated in patients with rapidly progressive early SOD1 fALS. Arimoclomol was administered orally; it has good bioavailability as it crosses the blood–brain barrier. Primary goal of the study was to assess safety and tolerability. Secondary outcome was efficacy, with main focus on survival. The rates of decline of the Revised ALS Functional Rating Scale (ALSFRS-R), percent predicted forced expiratory volume in 6 s (FEV6), and the Combined Assessment of Function and Survival (CAFS) were also used for efficacy evaluation (Benatar et al., 2018). Arimoclomol could treat a broad range of proteinopathies as the main action of this drug involves clearance of aberrant, misfolded, degraded, and aggregated protein by activation of HSPs.

#### Myricetin

The polyphenolic flavonoid, myricetin, has shown promise in targeting neurodegenerative diseases such as PD (Maher, 2019). The clear mechanism of action by which it upregulates proteasomal degradation mechanisms is not known. In ALS, cell culture studies have shown protein aggregate clearing effects of myricetin. Cos−7 cells were transfected with plasmid constructs of WT and mutant SOD1 leading to spontaneous intracellular accumulation of mutant SOD1 and WT accumulation after adding a proteasome inhibiting compound. On treatment with 10 μM myricetin for 48 h, immunofluorescence analysis showed a decrease in the intracellular aggregation of ubiquitin-positive SOD1. Myricetin increased chaperone HSP70 level and ultimately cell survival (Joshi et al., 2019).

#### Macrophage Migration Inhibitory Factor (MIF)

NSC-34 culture studies revealed that macrophage migration inhibitory factor (MIF) can reduce misfolded SOD1 and increase cell survival. One of the functions of MIF involves chaperone-like properties, which appear to change SOD1 amyloid aggregation pathways by forming disordered aggregates, which are less toxic to the cells (Shvil et al., 2018). Shvil et al. reported when NSC-34 cells are co-transfected with mutant SOD1-G93A and MIF, mutant SOD1 misfolding is affected by MIF, which prevents accumulation of mutant SOD1 in cytoplasm. MIF expression normalized the nuclear and cytoplasmic distribution of SOD1 to similar levels as wild-type SOD1 distribution (Shvil et al., 2018). Studies of MIF in animal models to investigate the therapeutic potential of MIF have not been performed.

### Targeting Aggregating Proteins by Suppressing Gene Expression

#### Superoxide Dismutase 1

Mutations in SOD1 cause 15–20% of fALS cases. The resulting amino-acid substitutions destabilize SOD1’s protein structure, leading to its self-assembly into neurotoxic oligomers and aggregates, a process hypothesized to cause MN degeneration. The aggregates are found in the brain and spinal cord of affected individuals as intracellular inclusions. The antisense molecule tofersen (BIIB067) was designed to bind SOD1 mRNA. The artificially created DNA specifically targets the mRNA stage. Tofersen prevents translation of mRNA and ultimately the RNA degrades due to abnormal DNA-RNA strands, thereby reducing mutant SOD1 protein production. A Phase 1 clinical trial assessing the safety, tolerability, and activity of tofersen in SOD1-related fALS patients has been completed. The randomized, double-blind, placebo-controlled safety trial tested four different doses of tofersen (0.15, 0.5, 1.5, or 3 mg) in 33 patients over a 12-h period. No serious adverse effects were observed in their assessment over 28 days post-treatment (Biogen, 2019). This treatment is moving to a Phase 3 trial to evaluate its efficacy in fALS. Another gene silencing approach that targets SOD1 by using adeno-associated virus (AAV) delivered shRNA in mice, pigs, and non-human primates showed prolonged suppression of MN disease. A new device design was used for injections which enabled homogeneous delivery throughout the cervical spinal cord white and gray matter and brain motor centers after a single subpial injection (Bravo-Hernandez et al., 2020). This approach could become an efficient strategy that may be extended to other delivery vectors.

#### Dipeptide Repeats of C9ORF72

The most common cause of fALS is the hexanucleotide repeat expansion in the C9ORF72 gene which is linked to 25–40% of all familial cases. Mutant C9ORF72 forms toxic DPRs (Gendron and Petrucelli, 2018). Recent studies have also demonstrated that arginine-rich DPRs (poly glycine-arginine/proline-arginine (GR/PR) are one of the major sources of neurotoxicity by targeting nucleopore complexes, thus affecting the nuclear–cytoplasmic trafficking of RNA and proteins (Zhang et al., 2015; Shi et al., 2018). Therefore, the gradual accumulation of those highly toxic DPRs, even at very low levels, could render neurons vulnerable (Lee et al., 2017). An experimental antisense oligonucleotide, BIIB078, developed by Ionis Pharmaceuticals, specifically targets C9ORF72 blocking its translation. This reduces the DPRs burden in the cell and their toxic effects. ALS mouse model studies showed extended survival and, surprisingly in some cases, muscle function also improved indicating reversal of disease symptoms. Phase 1 trials have been initiated focusing on assessing the safety of BIIB078. The trial will enroll nearly 60 people with ALS (Clinical Trials, 2019).

## Conclusion

A number of different compounds that target a diverse range of mechanisms of neuronal protein aggregation pathways have demonstrated early favorable results in preclinical and early clinical ALS studies. Those include targeting mRNA—both for degradation and to inhibit translation, clearance of protein aggregates using antibodies, disruption of protein–protein interactions that lead to aggregation, preventing the formation of aggregates using vaccines, and maintaining proteostasis by activating protein clearance mechanism. This diverse list of approaches highlights the complexity and the investigational challenges of targeting protein aggregation in ALS. Although there is growing evidence that the process of protein aggregation is an important driver of neurodegeneration, a proven structure–proteotoxicity relationship based on specific protein abnormalities such as folding or aggregate state is lacking. Further basic research into the role of protein abnormalities in ALS disease mechanism is needed to guide preclinical studies toward disease specific treatments.

## Author Contributions

RM contributed to writing the manuscript. MW contributed to writing and reviewing the manuscript.

## Conflict of Interest

The authors declare that the research was conducted in the absence of any commercial or financial relationships that could be construed as a potential conflict of interest.
